# The Therapeutic Alliance in Digital Mental Health Interventions for Serious Mental Illnesses: Narrative Review

**DOI:** 10.2196/17204

**Published:** 2020-08-07

**Authors:** Hailey Tremain, Carla McEnery, Kathryn Fletcher, Greg Murray

**Affiliations:** 1 Centre for Mental Health Faculty of Health, Arts and Design Swinburne University of Technology Hawthorn Australia; 2 Orygen Youth Mental Health Parkville Australia

**Keywords:** mental health, mHealth, eHealth, telehealth, psychosis, bipolar disorder, mobile phone

## Abstract

**Background:**

Digital mental health interventions offer unique advantages, and research indicates that these interventions are effective for a range of mental health concerns. Although these interventions are less established for individuals with serious mental illnesses, they demonstrate significant promise. A central consideration in traditional face-to-face therapies is the therapeutic alliance, whereas the nature of a digital therapeutic alliance and its relationship with outcomes requires further attention, particularly for individuals with serious mental illnesses.

**Objective:**

This narrative review aims to encourage further consideration and critical evaluation of the therapeutic alliance in digital mental health, specifically for individuals with serious mental illnesses.

**Methods:**

A narrative review was conducted by combining 3 main areas of the literature: the first examining the evidence for digital mental health interventions for serious mental illnesses, the second illuminating the nature and role of the therapeutic alliance in digital interventions, and the third surrounding practical considerations to enhance a digital therapeutic alliance.

**Results:**

Results indicated that a therapeutic alliance can be cultivated in digital interventions for those with serious mental illnesses, but that it may have unique, yet-to-be-confirmed characteristics in digital contexts. In addition, a therapeutic alliance appears to be less directly associated with outcomes in digital interventions than with those in face-to-face therapies. One possibility is that the digital therapeutic alliance is associated with increased engagement and adherence to digital interventions, through which it appears to influence outcomes. A number of design and implementation considerations may enhance the digital therapeutic alliance, including human support and technological features.

**Conclusions:**

More research is required to further understand the nature and specific role of a therapeutic alliance in digital interventions for serious mental illnesses, particularly in informing their design. This review revealed several key research priorities to advance the therapeutic alliance in digital interventions.

## Introduction

A therapeutic alliance is considered fundamental to the success of face-to-face psychological therapies but is an underrecognized consideration in digital mental health [1]. Given that digital mental health research and implementation is rapidly expanding, further exploration of the nature and role of a therapeutic alliance in this context is essential. The driving aim of this narrative review was to encourage further consideration and critical evaluation of a therapeutic alliance in digital mental health, particularly for serious mental illnesses. This review begins with a discussion of the current status of the literature regarding digital interventions and, specifically, those for serious mental illnesses. The following section highlights the relevance of a digital therapeutic alliance and the differences in the traditional conceptualization of therapeutic alliance. Next, the review focuses on conceptual issues surrounding a digital therapeutic alliance in the presence and absence of human support. Finally, practical considerations for enhancing digital therapeutic alliance are explored.

## Methods

To achieve the aims of this review, three areas of literature were integrated. The first was literature examining the potential of digital mental health interventions for serious mental illnesses, the second was literature illuminating the role of the therapeutic alliance in digital interventions, and the third providing practical considerations with the potential to enhance the digital therapeutic alliance. Digital mental health interventions can be defined as “interventions that provide information, support and therapy (emotional, decisional, behavioural and neurocognitive) for physical and/or mental health problems via a technological or digital platform” [2]. This paper will refer to mental health interventions with (supported) or without (unsupported) human support, accessed via mobile health or electronic health platforms. Given that the focus of this review was not contexts where a human delivers the majority of the intervention, including using digital means to deliver traditional face-to-face therapy sessions via the web or combinations of face-to-face and digital interventions (blended therapy), these were excluded where identified. The focus of this review was intervention studies with participants with a psychotic spectrum or bipolar disorder (BD) or any author-defined *serious mental illness*; however, where minimal data about serious mental illnesses are available, the broader mental health literature will be drawn upon.

## Results

### Potential Benefits of Digital Interventions

There are several obvious benefits of digital interventions to enhance mental health, such as overcoming logistical barriers and reducing therapeutic costs [3-5]. Indeed, some studies have suggested that these offer benefits not attainable through traditional therapy, such as the extension of the therapeutic hour, accessibility when and where users desire, and the provision of anonymity [4,6]. For these reasons, research into digital interventions is a burgeoning area, with many programs developed and evaluated in recent years.

### Specific Benefits of Digital Interventions for Serious Mental Illnesses

Perceived stigma, lack of insight, and lack of trust in available face-to-face treatments have been cited as key reasons for individuals with serious mental illnesses not seeking support for mental health concerns [7,8]. In addition, the symptoms and sequelae of BD and psychotic disorders, such as positive and negative symptoms, cognitive difficulties, general psychopathology, and poor social adjustment, are key predictors of poor adherence and engagement in face-to-face therapy [9-12]. Digital interventions offer the potential to address some of these specific barriers, for example, by increasing accessibility and a sense of autonomy and enabling self-pacing and reviewing of therapeutic content. Additional advantages include the provision of real-time and longitudinal patient data, potentially leading to a more accurate diagnosis and treatment decisions, moment-to-moment monitoring, and timely interventions [13]. Furthermore, digital interventions may be uniquely positioned to target the multilevel risk factors associated with poor physical health and mortality in serious mental illnesses [14]. Indeed, personal preference may mean that self-guided interventions are ideal for some [15].

#### Use of Digital Interventions

An additional layer of stigma that individuals with serious mental illnesses may experience is the assumption that they *cannot* or *do not* use digital devices for their mental health. A digital divide exists in mental health, wherein individuals experiencing serious mental illnesses are excluded from receiving support through digital means because of a lack of access, skills, or confidence [16]. However, evidence suggests that this exclusion is declining [17], particularly within psychotic disorders and BD [18,19]. However, developing digital interventions for individuals experiencing serious mental illnesses can introduce additional challenges because of illness-related factors, such as cognitive impairments or mistrust, thus requiring specific design considerations [12,16].

### Effectiveness of Digital Interventions

Overall, digital interventions for improving mental health have demonstrated significant promise. For example, 3 meta-analyses demonstrated that supported digital interventions for a range of mental health concerns were comparable in efficacy (moderate effect sizes) with face-to-face psychological interventions [20-22]. Currently, the greatest support exists for the effectiveness of digital interventions for depression and anxiety disorders, whereas data for other clinical disorders are fragmented at this stage. For example, a recent narrative review of digital interventions reported large effect sizes for participants with major depressive disorder, anxiety disorders, obsessive***-***compulsive disorder, and posttraumatic stress disorder and small effect sizes for eating disorders or problematic alcohol use [23].

#### Effectiveness of Digital Interventions for Serious Mental Illnesses

The findings of 6 recent systematic reviews of digital interventions for serious mental illnesses demonstrate that these are feasible and acceptable, with preliminary indications that these may be effective for symptoms and cognitive and social outcomes [24-29]. However, in each review, insufficient data were available to draw firm conclusions. Specifically, in a systematic review including individuals with psychosis, results provided preliminary evidence that these may have benefits for positive psychotic symptoms and depression, hospital admissions, medication adherence, socialization, and social connectedness [30]. For example, the recent Actissist proof-of-concept trial, in a small sample of individuals with early psychosis, demonstrated feasibility and acceptability through high use and adherence as well as symptom improvements in favor of the intervention group [31]. The handful of digital interventions for BD evaluated to date have produced inconsistent results. A systematic review of these studies showed that while these appear to be feasible and acceptable intervention options, inconsistent evidence was found for their effectiveness for symptoms or recovery outcomes such as quality of life [32]. The current Canadian Network for Mood and Anxiety Treatments and International Society for Bipolar Disorders treatment guidelines conclude that, to date, there is insufficient evidence to support the use of digital interventions as adjunctive therapies for BD [33]. However, multiple trials are underway, which will advance this literature [34-38].

### Therapeutic Alliance

The original conceptualization of the therapeutic alliance by Bordin [39] included 3 components: (1) the bond between the client and therapist, (2) agreement on the tasks directed toward improvement, and (3) agreement on therapeutic goals. This pantheoretical conceptualization has been widely adopted; however, there are various understandings of the therapeutic alliance. For example, Rogers [40] described the ideal qualities of a therapeutic relationship as acceptance, empathic understanding, and congruence, arguing that it is via these facilitative conditions that growth is achieved in therapy. An additional conceptualization, which forms the basis of the widely used Agnew Relationship Measure (ARM), comprises bond, partnership, confidence, openness, and client initiative [41].

#### Therapeutic Alliance and Outcomes in Face-to-Face Therapy

A number of meta-analyses point to a modest but reliable relationship between the quality of therapeutic alliance and outcomes of face-to-face therapy, with effect sizes typically in the moderate range [42-44]. Some research also supports the notion of a causal relationship between therapeutic alliance and outcomes in serious mental illnesses, for example, increasing the number of sessions attended was only beneficial in the presence of a strong therapeutic alliance for individuals experiencing psychosis [45]. Two systematic reviews focusing on psychosis found some evidence that the therapeutic alliance was related to reductions in psychotic symptoms, hospitalizations, and self-esteem outcomes [46] as well as functioning and treatment adherence [47]. Similarly, in BD, therapeutic alliance have been linked with decreased stigma, more positive attitudes toward medication, and, less conclusively, a reduction in symptoms [48].

#### Conceptualizing the Therapeutic Alliance in Digital Interventions

Given that the therapeutic relationship is a necessary (and some argue, sufficient) component of change in traditional face-to-face psychotherapies, a significant reservation toward digital interventions is the loss or, at a minimum, the modification of the therapeutic relationship. Some commenters have started to consider whether and, if so, how the therapeutic alliance plays a role in digital interventions [49]. Indeed, the James Lind Alliance identification of key priorities in advancing digital interventions included concerns relating to the therapeutic alliance in the top 10 issues raised [50]. The definition by Bordin has been transplanted to web-based environments, most commonly, digital interventions with human support components. However, it cannot be presumed that traditional conceptualizations of therapeutic alliance transfer to digital environments nor that different digital environments foster therapeutic alliance in equivalent ways.

The dimensions of the therapeutic alliance may differ in digital environments. A systematic review identified additional themes of *availability*, indicating how freely and conveniently accessible the digital intervention is, and *interactivity*, indicating the degree to which personalization and feedback based on user input is provided and to which the user feels in control [51]. These themes support the possibility of a perceived bidirectional relationship between a user and a digital system, in which automated aspects of the experience could emulate a reciprocal, trusted relationship. A qualitative analysis mirrored the importance of these domains within the therapeutic alliance for participants. For example, one participant identified that automated personalization helped them understood and conversely, another user would have perceived a *relationship* if the site responded intelligently [51]. Furthermore, following an examination of a supported digital intervention for carers, users perceived automated feedback, designed to emulate human communication, as supportive and helpful [52]. More work is needed to explore therapeutic alliance features and domains within digital contexts. Nonetheless, these investigations shed some light on the nature of the alliance in digital contexts, providing a *provisional characterization* of factors relevant to the alliance in digital contexts.

In parallel, the measurement of the therapeutic alliance requires specific consideration for digital environments. Simply replacing *therapist* with *program* or *app* in existing measures may fail to account for the complexity of the therapeutic alliance in digital interventions and parcel out the relative contributions of human-human and human-technology relationships. Researchers are beginning to acknowledge this; for example, WAI-Tech, a digital adaptation of the frequently used alliance instrument, the Working Alliance Inventory (WAI), includes reworded items in the *bond* subscale, omitting the human element [53]. Another adaptation applies to supported contexts, anchoring the task and goals subscales to the intervention and the bond subscale to the therapist [54]. Similarly, Berry et al [55] adapted the ARM for digital contexts by consulting end users and mental health professionals. In addition, the recently developed Enlight measure was specifically designed to assess the features of digital interventions, including those related to therapeutic alliance [56]. These measures require further validation but represent a starting point for capturing the unique qualities of the digital therapeutic alliance.

##### Therapeutic Alliance Ratings in Digital Interventions

Several recent reviews have demonstrated that client ratings of the therapeutic alliance in various digital interventions are of similar magnitude to those found in face-to-face therapies [42,57-59], including a small number of samples with serious mental illnesses [42]. Cavanagh et al [60] suggest that *common factors* cultivated within the face-to-face therapeutic alliance, such as hope, empowerment, credibility, expectancy, perspective, and emotional processing, might be achieved within digital interventions through a combination of human support components and the intervention itself, a triadic alliance.

##### Role of Human Supporters in Digital Interventions

Digital interventions vary with regard to their level of professional support, including no support (entirely self-managed interventions); minimal administrative or technical support; and tailored, regular support from a professional [61]. The frequency and type of support also ranges, but most frequently involve regular asynchronous message contact, typically with a professional, aimed at supporting participants’ engagement and progress through the program’s components [22]. Similarly, the content and model of support vary, and this may be an additional factor that impacts effectiveness and engagement [62].

A key issue when conceptualizing the therapeutic alliance in the digital context is whether this is dependent upon a relationship with a human supporter, and therefore whether a therapeutic alliance can be cultivated within wholly *unsupported* digital environments. In general, ratings of the therapeutic alliance are lower in unsupported interventions. For example, as shown in Multimedia Appendix 1 [53,63-78], 2 recent digital interventions for depression demonstrated positive ratings of the therapeutic alliance, which were lower than those in face-to-face versions [64,65]. On the other hand, qualitative interview participants largely rejected the idea of a relationship with the digital intervention, despite indications in their responses that they were experiencing alliance-like processes [51].

Digital interventions appear to be more effective when support is offered. The findings of multiple meta-analytic and systematic reviews suggest that digital interventions with support tend to have greater effect sizes than those without human support [21,79-83] for outcomes, including depression and anxiety symptoms, and general well-being. Several meta-analyses have shown that human support moderates the effectiveness of digital interventions [80,84]. Following a systematic review of factors influencing the successful implementation of digital interventions for those with serious mental illnesses, a key recommendation was the inclusion of human support elements [12].

In addition, supported digital interventions have comparably smaller attrition rates and greater engagement, as demonstrated in systematic reviews [23,85]. For example, a study evaluating predictors of adherence to digital interventions for individuals experiencing psychosis reported that support was associated with greater adherence [86]. Similarly, a recent study compared a digital intervention with and without support for individuals with a history of psychosis, finding that those receiving support engaged significantly more with the site, across a range of engagement parameters [87]. Furthermore, the inclusion of peer coaching within a digital intervention for BD significantly enhanced engagement and adherence [88].

However, it should be noted that some studies involving direct comparisons of supported and unsupported interventions have failed to demonstrate significant differences in effectiveness and/or adherence [89-91], with authors questioning whether a degree of human support is inherent under randomized controlled trial (RCT) conditions, while cautioning against interpreting these findings as evidence that support is not necessary. For example, Berger et al [92] found no differences in the effectiveness of a digital intervention for social anxiety across a range of clinical outcomes between unsupported, minimally supported, and flexibly (on-demand) supported programs; however, participants had access to peer support in this program, potentially providing relational benefits. More direct comparisons are required to draw definitive conclusions.

##### What Kind of Support and How Often?

Although it is evident that support is beneficial within digital interventions, little research has been conducted on the optimal form of support. A small group of studies have attempted to identify whether comparable benefits are attained with more versus less intensive (and, therefore, cost- and resource-effective) support. Klein et al [93] compared low (once weekly) and high (thrice weekly) frequency email support within a CBT digital intervention for panic disorder. Comparable symptom improvement, perceptions of a therapeutic alliance, and attrition were reported between conditions, suggesting that increased frequency of contact was neither a moderator of these outcomes nor essential to engagement. A recent review of digital interventions for psychosis similarly found that there was a little difference in adherence between interventions with low-, high-, or very high–frequency contact with supports [86].

Conversely, Palmqvist et al [82] reported a positive correlation between the amount of therapist contact in minutes and the effect sizes reported in 15 (supported and unsupported) digital interventions. A more recent systematic review of physical and mental health interventions with and without support reported that a higher frequency of support predicted better adherence [94]. One explanation for these contrasting findings is that the latter studies included unsupported interventions in their analyses, potentially skewing results, that is, these differences may reflect differences between no support versus support, rather than frequency. More research is required to unravel these contradictory findings and identify optimal levels of support and to examine these associations within serious mental illnesses.

Other potential influences on the impact of human support are the type of support or supporter. However, data to date do not support this conclusion. Lindner et al [95] found no differences in effectiveness between telephone and email support in a small trial. Baumeister et al [81] found no evidence that the qualifications of supporters influenced the outcomes in their systematic review. Similarly, the level of training did not impact the effectiveness of a digital intervention for depression [96]. Furthermore, a meta-analysis by Gellatly et al [84] failed to demonstrate any impact of the number of sessions, level of training, the content of the guidance, or mode of contact on therapeutic outcomes.

##### Potential Mechanisms

Support within digital interventions may be important for several reasons. For example, individuals with BD identified that managing procrastination and motivation issues were likely to be the primary benefits of support within a digital intervention [97]. Participants in an unsupported CBT digital intervention for depression echoed this, expressing the desire for support to assist with discipline and motivation [98]. Similarly, a meta-analysis of digital interventions for depression and anxiety disorders found that clients reporting lower motivation were less likely to benefit from interventions without support [61]. Furthermore, 2 qualitative analyses following digital interventions (for generalized anxiety disorder and depression) revealed that personalized support led to improved motivation to engage while fulfilling the needs for relatedness [99,100]. Therefore, a vital function of support within digital interventions may enhance motivation and a sense of relatedness.

#### Association of Therapeutic Alliance to Outcomes in Digital Interventions, With and Without Support

Although it has been established that the therapeutic alliance can be experienced in digital interventions, links to outcomes are less clear than in face-to-face therapies.

##### Supported Digital Interventions

A handful of studies on supported digital interventions have reported that ratings of therapeutic alliance are related to treatment outcomes. The first was a small secondary analysis from an RCT targeting anxiety disorders [67]. Ratings on a modified version of the WAI correlated with outcomes, specifically the degree of improvement in well-being and symptoms. In addition, Herbst et al [68] found that WAI ratings were associated with symptom reduction in a supported digital intervention for obsessive compulsive disorder (OCD). Following another OCD digital intervention in a larger sample, a therapeutic alliance was the best predictor of response to the intervention [69]. A systematic review aiming to examine whether a therapeutic alliance is associated with mental health outcomes found an association only within these 3 studies [58]. Two further studies demonstrated that, following small trials of a supported digital intervention for posttraumatic stress disorder (PTSD), ratings on the WAI were associated with reductions in PTSD symptoms [72,74].

Conversely, multiple studies have failed to find associations between the therapeutic alliance and treatment outcomes in supported digital interventions. For example, Andersson et al [70] found that high ratings on the WAI in 3 supported digital interventions did not correlate with change scores across outcome measures for depression, generalized anxiety disorder, and social anxiety disorder. Furthermore, Hadjistavropoulos et al [71] reported that ratings were unrelated to outcomes of supported digital interventions for either depression or generalized anxiety disorder. Similarly, Preschl et al [73] found that high WAI ratings, equivalent to the face-to-face comparison group, were not associated with treatment outcomes following a supported digital intervention for depression. Some quality issues should be noted here, specifically that these analyses were mostly secondary analyses with small samples and no controls, and, therefore, these findings require validation. In addition, studies have measured the alliance at different times (midtreatment [69,70], postintervention [68,73], or both [67,71,72]), whereas prior face-to-face research has indicated that the timing of alliance measurement influences its relationship with outcomes [101]. These inconsistencies may partly explain the discrepancies in the findings.

##### Unsupported Digital Interventions

In a small, open trial of an unsupported digital intervention, modified ARM ratings were lower than in face-to-face CBT and showed no associations with depression outcomes [65]. In another study, ratings on the WAI-Tech for a digital intervention were similar to those on the WAI in a face-to-face (treatment as usual) group but were not associated with the outcome: cocaine abstinence [53]. A further study found that ratings on the ARM were not correlated with outcomes for those with depression and anxiety, following an unsupported digital intervention [63]. As mentioned above, these are small studies, including an open trial [65] and secondary analyses [53,63], and the alliance was heterogeneously assessed, raising questions about reliability.

Taken together, there appears to be conflicting evidence that the therapeutic alliance links with outcomes in supported digital interventions, and no evidence of such an association is found in unsupported digital interventions. It appears that although the therapeutic relationship is robust to the reduced contact and distance in digital interventions, it may be less intimately tied to outcomes than in traditional therapies. Importantly, for this review, minimal data were available regarding these relationships for serious mental illnesses.

#### Association of Therapeutic Alliance With Engagement and Adherence in Digital Interventions, With and Without Support

Although evidence relating to a direct relationship between ratings of therapeutic alliance and treatment outcomes is inconclusive for digital interventions, one possibility is that the therapeutic alliance is associated with increased engagement and adherence, in turn, influencing outcomes [102]. It should be noted that engagement and adherence are often used interchangeably or poorly defined, limiting conclusions. In this review, engagement will refer to measures of the amount, frequency, or depth of program usage, whereas adherence will refer to whether participants met an a priori metric of intended usage or *dose*; however, we are limited to the information presented. For example, a real-world analysis of a range of apps and sites available to the public, with varying support, demonstrated that ratings of the therapeutic alliance (measured using the Enlight measure) were associated with increased user engagement [76].

##### Alliances and Engagement in Supported Interventions

Considering adherence, a study by Hargreaves et al [77] demonstrated that WAI ratings were the most significant predictor of adherence to a supported digital intervention for individuals experiencing psychosis. In another supported intervention, those that completed the intervention provided higher alliance ratings (in a sample with PTSD) [74]. A further study found a relationship between alliance ratings and engagement with intervention features in adolescents [78].

##### Alliances and Engagement in Unsupported Interventions

Clarke et al [63] did not find a direct relationship between therapeutic alliance (assessed with the ARM) and outcomes, while a significant association was discovered between the alliance and level of engagement with the unsupported digital intervention. In a further supported intervention, the WAI-Tech goal and bond subscales were positively correlated with the number of modules completed [53].

As can be seen, across the few studies that considered the possibility of a relationship between the alliance and engagement in digital interventions, all have reported an association. However, conclusions are complicated by the small number of studies and the range of conceptualizations of engagement and/or adherence therein.

#### Association of Engagement and Adherence to Outcomes in Digital Interventions

The above findings are especially compelling given that adherence and engagement are themselves associated with improved outcomes in digital interventions. For example, a meta-analysis demonstrated that adherence to both the intervention and the study was associated with a range of behavioral and health outcomes in digital interventions [103]. Furthermore, a systematic review showed that a broad range of *adherence* measures (many of which align with our definition of *engagement*) was associated with physical and psychological outcomes in digital interventions [104]. Associations with adherence have been demonstrated for a range of outcomes, including depression [105,106] and anxiety [107,108].

Engagement with digital interventions has also been linked to outcomes. Across outcomes such as depression and anxiety [109-111], greater changes in symptoms have been associated with better engagement with the site. Similarly, in a program for preventing eating disorders, the duration and extent of program use predicted some outcomes [112].

These findings suggest that a therapeutic alliance is associated with increased engagement with and adherence to digital interventions, in turn, influencing outcomes. However, this explanation represents one possible interpretation of these limited data and requires direct examination. Given to the inconsistencies within this literature [113], more work is needed to identify the engagement and adherence metrics with the most importance. In addition, no identified studies investigating links between outcomes, adherence, and engagement included samples with serious mental illnesses.

#### Conceptualizing the Therapeutic Alliance in Unsupported Digital Interventions

Although it appears that human support is beneficial for therapeutic alliance, in many cases, human support is not practical or desirable. Some users, including those with serious mental illnesses, report a preference for unsupported interventions, with benefits such as the reduced potential for judgment and increased honesty [25,51]. How the definition by Bordin [39] on therapeutic alliance can apply to unsupported interventions is not yet clear, but it has been suggested that therapeutic goals and therapeutic task components (or analogs thereof) may be cultivated within interactions between users and the intervention, while the *bond* element may be either entirely absent or modified [114]. For example, while equivalent ratings on the remaining alliance subscales were reported, the bond subscale of the WAI was significantly lower in a digital intervention for cocaine dependence than in a face-to-face group [53].

In the absence of human support, it may be more important to ensure that unsupported digital interventions incorporate automated features that resemble a bidirectional therapeutic relationship, such as the communication of empathy, responsiveness, and supporting the user’s motivation. Barazzone et al [115] examined the content of 3 widely used CBT programs to investigate the extent to which they incorporated key features for the establishment, development, and maintenance of a therapeutic alliance between the user and the program. They concluded that the programs exhibited substantial evidence of therapeutic alliance features, such as empathy and acceptance, and the negotiation of goals, providing feedback and building confidence in the program’s effectiveness, and rupture prevention and repair by encouraging users to return to the program after a break. Similarly, Holter et al [116] describe their attempts to cultivate a therapeutic alliance in a smoking cessation digital intervention, simulating a therapeutic alliance by allowing users to negotiate goals and using a conversational agent to implement *human* strategies such as empathy, interactivity (via *remembering* previous communications and tailoring), and humor. These authors did not assess the therapeutic alliance, its relationship with program features, or outcomes. However, Bickmore et al [66] evaluated the impact of introducing a relational agent that simulated alliance-promoting behaviors such as social dialogue, empathetic feedback, process comments, humor, and nonverbal communication, alongside a fitness digital intervention. The bond subscale was rated significantly higher within the group that had contact with the agent, whereas the overall WAI scores did not differ significantly.

Furthermore, additional *provisional characteristics* of the digital therapeutic alliance, availability, and interactivity, are not grounded in human support [51] and speak to the unique strengths of digital interventions. Technology is ideally positioned to be accessible when and wherever users require it, and a number of technological features can be optimized to cultivate interactivity, that is, a bidirectional relationship might be effectively emulated between a user and a digital intervention, with automated features. In addition, people may form attachments to smartphones, similar to an alliance [117,118], although users appear hesitant to call this a *relationship* [51]. Although human presence may be an important predictor of the therapeutic alliance in digital interventions, the *experience* of human presence may not require direct human contact. For example, the object-relations theory explains how devices may act as proxies for relationships with caregivers (the humans behind the intervention [119]).

Examining which technological features are best positioned to foster the therapeutic alliance in both supported and unsupported digital interventions is therefore an important next step, and we propose that exploring the additional provisional characteristics of availability and interactivity may represent a significant advancement.

### Persuasive Systems Design: Potential for Digital Therapeutic Alliance

**Figure 1:**
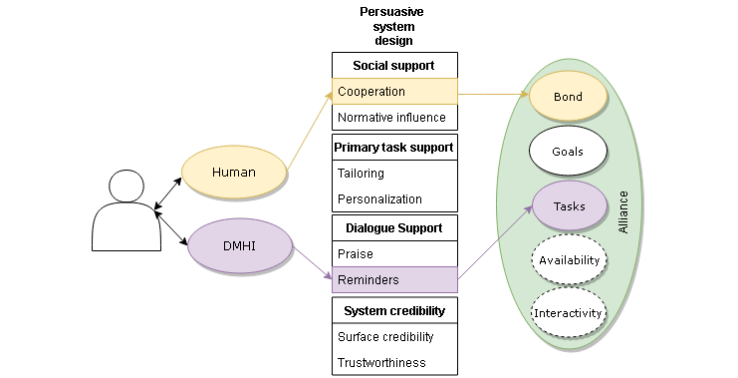
Representation of how specific persuasive systems design features could link to specific domains within the digital therapeutic alliance. DMHI: digital mental health intervention.

Particular consideration of the features of the intervention and their ability to promote behavior change [120] may provide direction for enhancing the therapeutic alliance. Persuasive systems design (PSD) is a broad term used to describe the technological features of a digital intervention specifically intended to motivate behavior or attitudinal change [94,121]. The study findings indicate that incorporating PSD features within interventions can be effective in motivating individuals toward specific goals across physical health [122,123], mental health [124,125], exercise [126,127], and educational [128] domains. The implementation of PSD with the specific intention of promoting the digital therapeutic alliance has not been examined to date; however, a number of PSD features [121] are likely to promote the development of a therapeutic alliance between users and digital platforms. This section provides an overview of the PSD factors that are likely to be relevant to the fostering of a therapeutic alliance in the digital space and comments on the potential role of human supporters, where applicable. Figure 1 presents an example of how aspects of the digital intervention itself and interaction with human supporters may promote specific, provisional components of the digital therapeutic alliance.

#### Tailoring and Personalization as Primary Task Supports

*Tailoring* systems and *personalizing* messages to users are important components of system design [121]. An example of tailoring is to suggest specific content based on what is known about users’ preferences or background, whereas personalization might reflect the person’s name or information about their medications, functioning, or previous responses [121].

Empirical research findings have indicated that when intervention content is matched with users’ psychosocial and behavioral characteristics, it is perceived as personally relevant, which enhances engagement [129] in line with the elaboration likelihood model [130]. Conversely, a 2016 review [85] indicated that the most commonly cited reason for low adherence within the digital intervention was a perception of impersonal or irrelevant content.

Therefore, tailoring and personalization may help foster a digital therapeutic alliance via congruency between the users’ personal needs and goals and interactions with the intervention. In addition, tailoring and personalization speak directly to the provisional digital therapeutic alliance’s characteristic of *interactivity*. For example, tailoring the intervention to a person’s stage of recovery in face-to-face therapy and not assuming *motivation* to change is predictive of a strong therapeutic alliance and outcomes [131]. In this vein, it has been argued that individually tailored content or responses within digital interventions enhance the sense of relationship with the platform, whether supported or unsupported [55]. Research findings provide some support for this claim, with qualitative accounts indicating that when users perceived an app as not tailored to their individual needs (eg, generic), the development of a relationship was hindered [55]. This is an important consideration when developing digital interventions for individuals with serious mental illnesses, where concerted efforts to build trust that the treatment will address the clients’ own unique goals may provide a buffer against feelings of mistrust or coercion [132].

#### Dialogue Support

*Dialogue support* is a PSD strategy that supports user-digital interactions deemed by users to be interpersonal or social interactions [121]. Specific examples of dialogue support in digital interventions include offering positive feedback (praise), reflecting users’ goals and tasks (reminders), linking users with peers via forums (social role), or providing recommendations for appropriate content (suggestions) [94,121]. Sustained engagement with a digital intervention is likely when the intervention offers ongoing interaction that is relevant, motivating, and tailored to users’ needs [113]. Review findings have demonstrated that adherence to digital interventions, in general, is contingent on the extent to which dialogue support elements are utilized [94]. Similarly, a recent review showed that a greater number of dialogue support elements was associated with beneficial outcomes [125].

Research findings also demonstrate that the quality of the therapeutic alliance is contingent on the perceived quality of the interaction and, therefore, implementing responsive dialogue support features within digital interventions should be a consideration in fostering a digital therapeutic alliance [133]. Improved dialogue between users and digital interventions could not only assist users to achieve their goals and foster a sense of interactivity but also enhance self-efficacy [121]. This is an important consideration for individuals with serious mental illnesses because research findings indicate that individuals who perceive greater control over their lives (ie, internal locus of control and greater self-efficacy) are more positive about the therapeutic alliance [134]. Furthermore, although it has not been studied in the context of digital interventions, an internal locus of control is associated with increased treatment motivation, compliance and treatment adherence, and better treatment outcomes in individuals with serious mental illnesses [135].

#### Credibility Support

*System credibility* or *credibility support* refers to how digital interventions incorporate technological features that convince the user that the system is credible [94,121]. Examples of how credibility support can be used in digital interventions include providing evidence-based content, expert moderation (eg, clinician support), and explicit, credible third-party endorsements [136]. Credibility support is associated with perceived authority, expertise, and trustworthiness among users of digital interventions, and findings show that its deliberate implementation is also associated with engagement [137].

Implementing credibility support features to increase users’ perceptions of expertise and trustworthiness is likely to help strengthen therapeutic alliance. For example, the bond between therapists and clients in face-to-face settings is contingent on the client’s confidence in the therapist’s competence [138]. Scant literature has examined this phenomenon within a digital context; however, Mackie et al [139] examined the effectiveness of a mobile-based intervention to treat harmful substance use in veterans presenting with self-harm and found that trust was associated with the quality of the digital therapeutic alliance, whereas damaged trust can lead to disengagement. This is a particularly important clinical consideration for individuals with serious mental illnesses who are often difficult to engage in ongoing treatment and have high dropout rates [132].

#### Social Support

Persuasive design strategies in the *social support* category refer to technological strategies that motivate users by leveraging social influence (eg, cooperation, social facilitation, and social comparison) [121]. Theoretically, individuals will be more motivated to perform a target behavior if they can use a system to observe (eg, social facilitation) or compare themselves (eg, social comparison) with others performing the behavior and if they are provided with a means to cooperate or connect [94,121]. An example of how digital interventions can include technological features to facilitate social support include incorporating newsfeeds and/or social forums. There is some support to suggest that the inclusion of social networking facilities in digital interventions for individuals with serious mental illnesses can assist in fostering a sense of social connectedness [35,140,141], a sense of cooperation (shared goals), and increased accountability toward treatment [137].

Developing digital interventions that utilize social support technological features may also help foster a digital therapeutic alliance. For example, study findings relating to face-to-face therapy have shown an association between clients’ levels of perceived social support and their ratings of the therapeutic alliance [142]. Research findings demonstrate that individuals with serious mental illnesses report benefits from interacting with peers on the web, including greater feelings of group belonging and social connectedness [143]. In addition, as discussed previously, individuals with serious mental illnesses who may be uncomfortable with or avoidant of conventional social contact may perceive digital interactions as less threatening [144]. Therefore, the facilitation of social support should be a design feature of digital interventions for individuals with serious mental illnesses.

## Discussion

### Implications

There are a number of implications of these findings for the development and evaluation of digital interventions, specifically for designing interventions for individuals with serious mental illnesses. Specifically, there have been very few investigations into the role of specific design features in cultivating digital alliances. For example, if *interactivity* is confirmed as an important feature of the digital alliance, some specific design principles (such as tailoring and personalization strategies) are ideally positioned to promote users’ perceptions of interactivity. Furthermore, the unique nature of the alliance in digital contexts is likely to interact specifically with serious mental illnesses. For example, individuals who may be uncomfortable with or encounter barriers to interacting within traditional therapeutic encounters may be differentially receptive to forming relationships in digital contexts (even those without human support), and as noted, some individuals prefer accessing digital interventions over traditional forms of support.

### Limitations

The main limitations of this review were that no systematic search methods or formal bias and quality assessment methods were used, as the aim was a broad integration of relevant literature to identify key gaps for future research. Accordingly, there is a significant risk of selection bias. Therefore, the included studies cannot be presumed to represent the available literature, and firm conclusions are not warranted on these bases. In addition, in many included studies, the analyses that were included were secondary analyses (Multimedia Appendix 1), and many were underpowered because of sample size, inflating type I and type II error risk. There may also be some conceptual issues impacting the results. For example, issues unique to the experiences of individuals with serious mental illnesses and their potential interactions with the relationships of interest should be considered. For example, social withdrawal, mistrust, or paranoia could interact with engagement and independently with an alliance.

### Future Directions

More research is required to further understand the specific role of the therapeutic alliance in digital interventions for serious mental illnesses, particularly in informing their design. Specifically, based on the findings of this review, systematic reviews could examine the role of the therapeutic alliance in both engagement and outcomes of digital interventions. Furthermore, a great deal of research has provided data about the role of human support, largely indicating that it is beneficial (but not essential). However, further inquiry is needed to identify the optimal form, intensity, and role that should be taken and to examine nuances such as interactions with different presentations or populations, including those with serious mental illnesses. Separate to this, ways of maximizing therapeutic alliance that are independent of human support require further investigation, and new technologies present multiple promising avenues. The potential for PSD features to enhance the digital therapeutic alliance requires further investigation. Digital phenotyping and machine learning approaches, integrating physiological and behavioral signals to tailor interventions with the aim of enhancing outcomes, demonstrate promise, and for serious mental illnesses, the research in this area is growing [13,145-147]. However, the specific role of such technologies in cultivating digital therapeutic alliance remains to be unknown. Virtual reality (VR) is another area of growth in mental health; however, limitations to date inhibit the potential for exploring its impact on the therapeutic alliance, including that most VR settings require face-to-face contact with a human, and these technologies currently offer the minimal ability for interpersonal interactions within virtual environments [148]. Research on the role of a therapeutic alliance in VR is limited, with preliminary studies showing that therapeutic alliance can be cultivated with these technologies [149]. An additional line of research requiring further attention is concerned with the way digital interventions might be integrated with or enhance care models, such as stepped care, blended models, and virtual clinics, each with specific implications upon a therapeutic alliance [150].

### Conclusions

Digital mental health interventions continue to garner significant research attention, demonstrating effectiveness for a range of mental health outcomes and holding significant promise for those with serious mental illnesses. Although a long tradition of research has demonstrated that the therapeutic alliance is central to outcomes in face-to-face therapy, less emphasis has been placed on the therapeutic alliance in digital contexts. Overall, it appears that a therapeutic alliance can be cultivated in digital interventions, but it may have unique, yet to be confirmed, features in these contexts. With further investigation, it may emerge that the nature of the alliance is so divergent as to reflect a unique construct; however, at this juncture, we believe it is reasonable to consider relationships cultivated in digital context analogs of traditional, bidirectional therapeutic alliance, and hence, this term is used throughout this work. In addition, it appears likely that the therapeutic alliance is less directly linked with outcomes in digital interventions than in face-to-face therapies. Rather, it may be that the therapeutic alliance is associated with increased engagement with and adherence to digital interventions, which, in turn, leads to improved outcomes. Human support is an effective method of enhancing both engagement and therapeutic alliance; however, this is not always feasible or desirable. Alternatively, alongside or in lieu of human support, technological features may be capable of cultivating therapeutic alliance, especially when these are automated to emulate relational characteristics, but more research is needed to explore which features are most closely aligned with the improved therapeutic alliance and, ultimately, improved outcomes. Accordingly, several research priorities have been identified to advance this understanding.

## Appendix

Multimedia Appendix 1 Intervention characteristics and reported links between alliance, engagement, and outcomes.

## Abbreviations

ARM: Agnew Relationship Measure
BD: bipolar disorder
OCD: obsessive compulsive disorder
PSD: persuasive systems design
PTSD: posttraumatic stress disorder
RCT: randomized controlled trial
VR: virtual reality
WAI: Working Alliance Inventory
