# Supplementary material for: The Therapeutic Alliance in Digital Mental Health Interventions for Serious Mental Illnesses: Narrative Review
Source: JMIR Ment Health. 2020 Aug 7;7(8):e17204. doi: 10.2196/17204 (PMC7442952; doi:10.2196/17204)
Supplement: Multimedia Appendix 1 [file mental_v7i8e17204_app1.pdf]

Table 1. Intervention characteristics and reported links between alliance, engagement, and outcomes.

Author	Design and intervention (control)	Sample characteristics	Therapeutic alliance characteristics	Association with outcomes	Association with engagement or adherence
Gega et al [64]	• Case series<br>• Unsupported computerized CBT[a] (therapist-delivered CBT)	• 6 participants with symptoms of depression (2 women, age *mean* 25 years[b])	• *Session Evaluation Questionnaire* was lower for computerized CBT than F2F[c] for all participants<br>• *Session Impact Scale* score showed that those who completed therapy rated cCBT lower than F2F for developing understanding and building a therapeutic relationship	—[d]	—
Bickmore et al [66]	• Computerized exercise program with an embodied relational	• 91 healthy adults with a desire to increase exercise (50 women; *mean*	• Higher ratings were provided on the bond subscale of an adapted WAI[e]	—	

	agent—a computer-animated avatar demonstrating complex verbal and nonverbal behaviors aimed at developing an alliance (no agent versus nonrelational agent)	age 24.8 [*SD* 7.4] years)	with increases over time, when the relational agent was used.		
Ormrod et al [65]	• Pilot, open trial<br>• Supported computerized CBT (none)	• 23 adults with depression (12 women; *mean* age 47 [*SD* 11] years)	• Positive therapeutic alliance ratings (on a modified version of the *ARM*[f]), which were lower across all scales than those in F2F therapy	• In the *ARM*, the total alliance ratings were not associated with changes to depression. No significant relationship was found between any subscale of the *ARM* and	—

				depression outcomes measured	
Bergman Nordgren et al [67]	• Secondary analysis from an RCT[g] • Supported web-based CBT (online discussion forum; data not used in this analysis)	• 27 participants with anxiety disorders (18 women; *mean* age 39 [*SD* 11] years)	• High working alliance ratings (*WAI*), which increased from pre- and mid- to posttreatment	• Total *WAI* alliance ratings mid- and posttreatment (but not pretreatment) correlated with outcomes, specifically, the degree of improvement to well-being and symptoms • Subscale scores not assessed	—
Herbst et al [68]	• Secondary analysis from an RCT • Supported web-based CBT (waitlist control, but in this analysis, their data was	• 30 participants with OCD[h] (22 women; age 19-59 years)	• High *WAI* ratings on all subscales. Assessed at intervention endpoint only	• Total *WAI* ratings were associated with symptom reduction • Not assessed on the subscale level	—

	included after receiving the intervention)				
Andersson et al [69]	• Secondary analysis from RCTs<br>• Supported web-based CBT (waitlist control, but in this analysis, their data were included after receiving the intervention)	• 101 adults with OCD (67 women; *mean* age 34.9 [*SD* 12.7] years)	• Total WAI assessed was adequate	• Total *WAI* was associated with symptom change and was the best of the assessed predictors of response to the intervention (especially at higher severity)<br>• Not assessed on the subscale level	—
Andersson et al [70]	• Secondary analysis of data from 3 controlled trials<br>• Supported web-based CBT (with email and waitlist	• Sample 1: 49 depressed adults (37 women; mean age 39 [SD 13.5] years)<br>• Sample 2: 35 adults with a	• High alliance (*WAI*) in all samples, comparable with previously reported F2F ratings—measured	• The association between alliance ratings (total or subscale scores), and the outcome was	—

	controls, whose data were not analyzed here)	generalized anxiety disorder (28 women; mean age 40 [SD 11.2] years)<br>• Sample 3: 90 adults with a social anxiety disorder (54 women; mean age 38.0 [SD 11.4] years)	*early* in treatment	nonsignificant across all 3 samples	
Hadjistavropoulos et al [71]	• Secondary analysis from an open dissemination study of a supported web-based CBT	• Adults with symptoms of depression (n=83) or generalized anxiety (n=112; 135 women; *mean* age 40.2 *[SD* 12.6] years)	• *Therapeutic Alliance Questionnaire* ratings were high in both samples, at mid- and endpoints (and did not differ between samples)	• Ratings on the *Therapeutic Alliance Questionnaire* were unrelated to outcomes (but correlated with treatment satisfaction)	—
Knaevelsrud et al [72]	• RCT<br>• Supported, web-based CBT	• 96 adults with a history of trauma and posttraumatic	• High alliance rated on the *WAI* by participants and therapists.	• Ratings on the *WAI* posttreatment were	—

	(waitlist control)	stress reactions (86 women; *mean* age 35 years)	Participants' ratings of the working alliance (total) significantly improved during treatment	associated with outcomes	
Preschl et al [73]	• RCT<br>• Supported, web-based CBT (F2F CBT)	• 53 adults with depression (36 women; *mean* age 36.7 [*SD* 10.9] years)	• High WAI ratings, total and across all subscales, equivalent to the F2F comparison group	• *WAI* ratings did not predict a change in depression scores (neither total alliance nor subscale)	
Clarke et al [52]	• Secondary analysis of RCT data<br>• Unsupported web-based CBT (TAU[i] control, data not used in this analysis)	• 90 adults with mild-to-moderate depression, anxiety, or stress who had completed the intervention (65 women; *mean* age 38 [*SD* 10] years)	• Positive alliance ratings on all *ARM* subscales	• No association between *ARM* total or subscales alliance and functioning, well-being, or symptoms	• The 3 relationship-focused domains of the *ARM* were associated with all engagement metrics
Wagner et al [74]	• Secondary analysis from an RCT	• 55 adults with posttraumatic stress	• High *WAI* scores early in treatment	• Total working alliance predicted	• Slightly but nonsignificantly higher

	• Supported, web-based CBT (waitlist control, but in this analysis, their data were included after receiving the intervention)	symptoms (43 women; *mean* age 27.7 [*SD* 6.9] years)	were maintained for the duration, across all subscales	treatment outcomes for posttraumatic stress symptoms. On the subscale level, symptom change midtreatment was correlated with the midtreatment task subscale score and the posttreatment task and goal subscale scores<br>• Furthermore, posttreatment symptom change scores were correlated	*WAI* scores for completers, on all subscales

				with midtreatment task and bond and posttreatment goal and task subscale scores	
Kiluk et al [54]	• Unsupported web-based CBT (TAU CBT)	• 66 cocaine-dependent adults (33 women; *mean age* 42.3 [*SD* 9.6])	• *WAI-Tech* scores for the digital intervention were similar to those on the WAI, however scores on the bond subscale were lower than in a F2F (treatment as usual) group	• *WAI-Tech* ratings were not associated with outcomes (cocaine abstinence) in the digital group, but total WAI was associated with outcomes in the F2F group	• *WAI-Tech* goal and bond subscales at session 4 were positively correlated with the number of modules completed
Baumel et al [75]	• 30 real-world electronic health programs (preliminary study for	• 30 programs (median 110 users each)	• *Enlight* measure: mean scores on therapeutic alliance and therapeutic persuasiveness	—	• One aspect of the alliance, therapeutic persuasiveness, predicted engagement

	Baumel et al [76], varying support)		scales were in the poor to fair range		whereas alliance scale did not. However, therapeutic persuasiveness contains components that are aligned with the alliance (eg, interactivity)
Baumel et al [76]	• 52 mobile apps and 32 websites	• App downloads: median 38,600<br>• Websites monthly unique visitors: median 5689	• *Enlight* measure median scores on therapeutic alliance and therapeutic persuasiveness scales were in the poor to fair range	—	• Total and subscales of the alliance were associated with engagement metrics<br>• Total and some elements of therapeutic persuasiveness were associated with engagement metrics, both predicted

					total time engaged
Hargreaves, et al. [77]	• Computerised cognitive remediation with F2F support (open conversation with therapist, data not included here).	• 48 participants with a history of psychosis and subjective or objective cognitive difficulties (31 women, age *M* = 43.5, *SD* = 11.5)	• **WAI** – scores not disclosed.	-	• **WAI** ratings were the most significant predictor of adherence; **WAI** moderately correlated with minutes of intervention completed.
Anderson, et al. [78]	Secondary analyses of an RCT. Study 1: Supported web-based CBT (F2F CBT) • Study 2: Supported web-based CBT	Sample 1: 73 adolescents (12-18 years) with an anxiety disorder and their parent (45 female, age *M* = 13.9, SD = 1.6) • Sample 2: 132 children or adolescents (7-18 years) with an anxiety disorder and their parent *including the intervention group from*	• Adolescents reported equivalent **WAI** scores, reflecting strong alliance, in F2F and web-based, parents reported slightly higher scores in F2F	For adolescents (but not children or the total sample) there was a significant relationship between alliance (both youth and parent rated) and change in outcomes.	Alliance predicted intervention compliance (engagement). Compliance did not mediate the relationship between alliance and outcomes.

		study 1 (70 female, age *M* = 12.1, *SD* = 2.5)			

[a]CBT: cognitive behavioral therapy.

[b]Means and standard deviations for participants’ age were provided wherever this was available within the original article.

[c]F2F: face-to-face.

[d]Em dash indicates that this article did not provide relevant data for this cell.

[e]WAI: Working Alliance Inventory.

[f]ARM: Agnew Relationship Measure.

[g]RCT: Randomized controlled trial

[h]OCD: Obsessive Compulsive Disorder

[i]TAU:Treatment as Usual

References

64. Gega L, Smith J, Reynolds S. Cognitive behaviour therapy (cbt) for depression by computer vs. Therapist: Patient experiences and therapeutic processes. *Psychother Res.* 2013;23(2):218-231.PMID:23390994

66. Bickmore T, Gruber A, Picard R. Establishing the computer-patient working alliance in automated health behavior change interventions. *Patient Educ Couns.* 2005;59(1):21-30.PMID:16198215

65. Ormrod JA, Kennedy L, Scott J, Cavanagh K. Computerised cognitive behavioural therapy in an adult mental health service: A pilot study of outcomes and alliance. *Cogn Behav Ther.* 2010;39(3):188-192.PMID:20485996

67. Bergman Nordgren L, Carlbring P, Linna E, Andersson G. Role of the working alliance on treatment outcome in tailored internet-based cognitive behavioural therapy for anxiety disorders: Randomized controlled pilot trial. *JMIR Res Protoc.* 2013;2(1):e4.PMID:23612437

68. Herbst N, Franzen G, Voderholzer U, et al. Working alliance in internet-based cognitive-behavioral therapy for obsessive-compulsive disorder. *Psychother Psychosom.* 2016;85(2):117-118.PMID:26807976

69. Andersson E, Ljótsson B, Hedman E, et al. Predictors and moderators of internet-based cognitive behavior therapy for obsessive–compulsive disorder: Results from a randomized trial. *Journal of Obsessive-Compulsive and Related Disorders.* 2015;4:1-7

70. Andersson G, Paxling B, Wiwe M, et al. Therapeutic alliance in guided internet-delivered cognitive behavioural treatment of depression, generalized anxiety disorder and social anxiety disorder. *Behav Res Ther.* 2012;50(9):544-550.PMID:22728647

71. Hadjistavropoulos HD, Pugh NE, Hesser H, Andersson G. Therapeutic alliance in internet-delivered cognitive behaviour therapy for depression or generalized anxiety. *Clin Psychol Psychother.* 2017;24(2):451-461.PMID:27060617

72. Knaevelsrud C, Maercker A. Internet-based treatment for ptsd reduces distress and facilitates the development of a strong therapeutic alliance: A randomized controlled clinical trial. *BMC Psychiatry.* 2007;7:13.PMID:17442125

73. Preschl B, Maercker A, Wagner B. The working alliance in a randomized controlled trial comparing online with face-to-face cognitive-behavioral therapy for depression. *BMC Psychiatry.* 2011;11(1):189.PMID:22145768

52. Clarke J, Proudfoot J, Whitton A, et al. Therapeutic alliance with a fully automated mobile phone and web-based intervention: Secondary analysis of a randomized controlled trial. *JMIR Ment Health.* 2016;3(1):e10.PMID:26917096

74. Wagner B, Brand J, Schulz W, Knaevelsrud C. Online working alliance predicts treatment outcome for posttraumatic stress symptoms in a rab war-traumatized patients. *Depress Anxiety.* 2012;29(7):646-651

54. Kiluk BD, Serafini K, Frankforter T, Nich C, Carroll KM. Only connect: The working alliance in computer-based cognitive behavioral therapy. *Behav Res Ther.* 2014;63:139-146.PMID:25461789

75. Baumel A, Yom-Tov E. Predicting user adherence to behavioral ehealth interventions in the real world: Examining which aspects of intervention design matter most. *Transl Behav Med.* 2018;8(5):793-798.PMID:29471424

76. Baumel A, Kane JM. Examining predictors of real-world user engagement with self-guided ehealth interventions: Analysis of mobile apps and websites using a novel dataset. *J Med Internet Res.* 2018;20(12):e11491.PMID:30552077

77. Hargreaves A, Dillon R, Castorina M, et al. Predictors of adherence to low support, computerised, cognitive remediation training in psychosis. *Psychosis.* 2018;10(4):298-306.PMID:WOS:000452285700006

78. Anderson RE, Spence SH, Donovan CL, March S, Prosser S, Kenardy J. Working alliance in online cognitive behavior therapy for anxiety disorders in youth: Comparison with clinic delivery and its role in predicting outcome. *J Med Internet Res.* 2012;14(3):e88.PMID:22789657TAU
